# Impact of Wagering Inducements on the Gambling Behaviors, Cognitions, and Emotions of Online Gamblers: A Randomized Controlled Study

**DOI:** 10.3389/fpsyt.2020.593789

**Published:** 2020-11-17

**Authors:** Gaëlle Challet-Bouju, Marie Grall-Bronnec, Anaïs Saillard, Juliette Leboucher, Yann Donnio, Morgane Péré, Julie Caillon

**Affiliations:** ^1^CHU Nantes, Addictology and Psychiatry Department, Nantes, France; ^2^Nantes University, Tours University, INSERM, SPHERE U1246 ≪ MethodS in Patients-centered outcomes and HEalth ResEarch ≫, Nantes, France; ^3^CHU Nantes, Biostatistics and Methodology Unit, Department of Clinical Research and Innovation, Nantes, France

**Keywords:** online gambling, wagering inducement, gambling expectancies, loss of control, gambling disorder, responsible gambling, addiction, prevention

## Abstract

Wagering inducements are part of loyalty/reward programs implemented by online gambling operators to retain or attract consumers. They constitute incentives to bet that are offered to gamblers provided that they perform certain betting-related activities. They are often considered risk factors for gambling problems, but studies exploring the actual impacts of such incentives are scarce. The objective of the present study was to assess the actual impact of wagering inducements on gambling behaviors, cognitions, and emotions of online gamblers. One hundred seventy-one adults (18–65 years old) who gamble on a regular basis on the Internet, including at-risk and recreational gamblers, were recruited through media announcements and in panels from survey institutes. Participants were randomly assigned to one of four experimental conditions, in which a defined amount of money was given to the gambler with a bank e-card system during an experimental gambling session to simulate a wagering inducement (€10, €50, €100, or €200), or the control condition, in which no incentive was given. The experimental gambling session was designed to be as natural as possible (participants gambled with their own gambling account and their own money). Participants completed a pretest interview, took part in the experimental gambling session, and then completed a post-test interview. The impact of wagering inducements was estimated on objective (money wagered and time spent gambling during the gambling session) and subjective (cognitive distortions, enjoyment of gambling, loss of control, and respect of usual gambling habits) gambling endpoints that were compared between conditions. Two-thirds of participants reported having already received wagering inducements at some point of their gambling course. Although no effect was demonstrated on time spent gambling, inducements increased money wagered, gambling-related expectancies and perceived loss of control. In particular, it seems that wagering inducements could lead to extreme expenses, especially for at-risk gamblers. This research suggests that regulating wagering inducements could be helpful for prevention and early intervention. Future research on the impacts of wagering inducements is still needed, especially more ecological studies based on behavioral tracking data and studies assessing the differential impacts of various incentive types.

**Clinical Trial Registration:** NCT01789580 (ClinicalTrials.gov).

## Introduction

Gambling problems concern only a minority of gamblers (from 0.1 to 5.8%, depending on country) (1). However, the Internet was identified as a risk factor for problem gambling due to its high accessibility, anonymity, high frequency of gambling outcomes, and digital payment modes (2–4). Gambling on the Internet leads to higher risk for and higher severity of gambling problems for online gamblers compared to offline gamblers (5–9). For example, in France, where Internet gambling was legalized in 2010, the prevalence of past-year gambling problems has continuously increased since 2010 to reach 6% of past-year gamblers in 2019 (10), with Internet gamblers being twice as likely to be excessive gamblers (11). Indeed, a survey conducted exclusively on Internet gamblers revealed a prevalence of gambling problems of 22.4% in 2017 (12).

As for other markets, gambling operators implement marketing strategies to boost sales and generate loyalty among their customers (13). In particular, the use of loyalty/reward programs is widespread among online gambling operators (13). Such marketing strategies include both loyalty programs and instant reward programs. Loyalty programs include incentives that are given to consumers in response to repeated consumption and are expected to reinforce consumption in those consumers in the long term (14, 15). In contrast, instant reward programs are short-term programs including one-off advantages that reward consumers instantly with incentives (15, 16). In the framework of gambling, instant reward programs include wagering inducements, presented as sales promotions. According to Hing et al., wagering inducements are defined as incentives to bet that are given to gamblers in addition to what is normally received as part of the core wagering product; wagering inducements are conditional upon certain betting-related activities and/or redeemed in a form that encourages betting and aim to trigger specific consumer responses (such as inducing an immediate sale, retaining consumers, prompt brand switching, and intensify purchasing) (17). Wagering inducements can take different forms, such as sign-up and referral offers, matching deposits with bonus bets, refund/stake back offers, and bonus or better odds (17). They may also vary according to the type of gambling (race or sports betting, poker, lotteries, etc.). In the French online gambling market, wagering inducements are very common and represented 179 million euros during 2019 for online sports betting, horse betting and poker alone (18).

Little research has been performed on loyalty programs in the framework of gambling (13, 15). For example, a report commissioned by Gambling Research Australia in 2014 found only 16 articles about loyalty programs specific to the gambling industry, which were exclusively focused on casinos, and none from France (15). The large majority of articles on loyalty programs in gambling are from a marketing perspective (15). As an illustration, only one qualitative study identified by the report commissioned by Gambling Research Australia explored the impact of loyalty programs on vulnerable and at-risk gamblers (15). This study explored the way in which gamblers interpret and respond to marketing strategies, including incentives (19). Gamblers were influenced by incentives in various ways, mainly depending on their age and sex. Older women without problem gambling appreciated the social benefits and free meals offered through incentives but were realistic about the associated risks. In contrast, younger men, gamblers with low socioeconomic backgrounds and problem gamblers mainly focused on the benefits associated with the incentives but did not take into account their long-term risks. They described the impact on their gambling behavior (shift toward online activities, opening new accounts, or gambling on multiple websites) but considered incentives as “no lose” benefits, especially for problem gamblers. However, a recent study has demonstrated that gamblers tend to underestimate the true cost of bonus bets (20). Indeed, bonus bets often have conditions of use that imply additional gambling expenditures from gamblers, which are not always clearly stated in advertisements and, by extension, understood by gamblers. This may lead one to question the principle of informed choice for responsible gambling consumption. In a qualitative study performed in 2014, the same team reported increased gambling in response to bonus offers in treatment-seekers but not in general population gamblers, with treatment-seekers feeling strongly tempted to drop resolutions of controlled gambling (21). Another qualitative study on sports betters, also conducted in Australia, indicated that incentives such as bonus bets were considered by gamblers among the most effective marketing strategies (22). They conceptualized these incentives as safety bets or free money, which led them to open multiple accounts (for long-term use rather than short-term use as initially intended), gamble at moments when they would normally not do so, and feel greater control over their gambling, even if they are aware that inducements are a marketing strategy. An online survey of 1,813 sports bettors also reported that the consumption of wagering inducements may lead to impulsive in-play betting patterns, especially among problem gamblers, and frequent sports viewers (23). More recently, another online survey from the same team highlighted the impact of incentives, including bonus bets, on increasing risk taking in a simulation of sports betting (24).

Those studies, while being very instructive on the potential impact of wagering inducements, relied mainly on qualitative designs, self-reported data or online surveys, which suffer from poor ecological validity. A recent study on the impact of exposure to inducements on betting behaviors included an ecological momentary assessment design with higher ecological validity (25). In this study, almost 600 gamblers completed up to 15 ecological momentary assessments to report their exposure to different types of wagering advertisements and inducements, along with intended and actual betting expenditure. The results indicated that wagering inducements and advertisements were associated with more frequent and more intense betting. However, such a design may suffer from problems of chronology because of the dynamic interrelation between closely interrelated outcomes that may influence each other over time; that is, the attribution of wagering inducements depends on previous gambling behaviors, and gambling behaviors may be influenced by inducements, the latter being the causal dynamic of interest from a gambling prevention perspective. The authors of this study thus recommended measuring betting behavior that occurs strictly after exposure to inducements to capture a causal interpretation.

The objective of the present study was thus to assess the actual impact of wagering inducements on gambling behaviors in experimental research with both objective (money wagered, time spent gambling) and subjective (cognitions, emotions) gambling endpoints and high ecological validity (real money, real gambling websites). We hypothesized that wagering inducements would lead to increased gambling behavior and gambling-related cognitions and emotions during a gambling session. More specifically, we made the assumption that the impact of wagering inducements would be stronger for participants at risk for gambling disorder compared to low-risk controls, and would vary depending on preferred gambling activities. This hypothesis was tested by manipulating wagering inducements in a sample composed of at-risk gamblers and recreational gamblers with various favorite types of gambling activity, and monitoring betting behavior during an experimental gambling session in which participants were able to wager on their own preferred websites.

## Materials and Methods

The present work is part of the MOD&JEU research program (trial registration number: NCT01789580), previously described in a study protocol available at https://www.ncbi.nlm.nih.gov/pmc/articles/PMC4448208/ (26). The MOD&JEU research program is composed of four randomized controlled studies assessing the effectiveness of various types of Internet gambling protection tools [self-limitation, pop-up messages, and self-exclusion (27)] and the impact of wagering inducements on gambling behaviors. The present work reports the results of the study dedicated to wagering inducements.

### Participants

Participants were volunteers gambling regularly and currently on the Internet. To represent a wide range of gambling profiles in the sample, half of volunteers were at-risk gamblers, and half were recreational gamblers [according to the scoring of the Problem Gambling Severity Index (PGSI) (28)].

The inclusion criteria were as follows: only adults (18–65 years old) gambling at least once per month on a licensed French website who agreed to give the research team access to their gambling account information (to provide access to their gambling history, making it possible for the research team to objectively collect data on changes in gambling behavior during the period of interest) and who have set their deposit limit to at least €200 (in order to be able to implement any of the experimental conditions set out in the study). Non-inclusion criteria were being a problem gambler according to the PGSI (scoring 8 or more), being currently treated for a gambling problem, being indebted, being pregnant, being under protective measures (guardianship or curatorship), having used psychoactive substances on the day of the experiment, participating in another clinical study during the week preceding the experiment, and having a history of psychosis or severe cognitive impairment. Problem and treated gamblers were not included to prevent the amplification of their gambling problems by putting them in a gambling situation as part as this research.

### Procedure

The recruitment took place between March 2013 and February 2018. Participants were recruited through media announcements (newspapers, radio, and websites) and in panels from survey institutes. Volunteers were requested to contact the research team by email to obtain details on the study and arrange a telephone appointment to complete the pre-selection questionnaire to assess eligibility. Eligible participants were invited to come to the research center for a half day to perform the research procedure.

First, participants were randomly assigned to one study from the MOD&JEU research program (wagering inducements, self-limitation, pop-up messages, or self-exclusion) and then to one of the experimental conditions of the assigned study. In the case of the wagering inducements study, participants were randomly assigned to four experimental conditions (€10, €50, €100, or €200, with an expected sample size of 30 each) or the control condition [expected sample size of 60, calculated as 30^*^√(k), where k was the number of experimental conditions]. The graduated amount of money used for the experimental conditions was chosen to estimate the impact of wagering inducements depending on the amount of money. The randomization was stratified according to the gambler's status (recreational or at-risk) and to the favorite type of gambling activity (pure chance games: lottery and scratch cards; skill and chance bank games: horserace and sports betting; and skill and chance social games: poker).

All participants completed a pretest interview prior to the experiment to provide the following information: sociodemographic characteristics, previous knowledge, use and opinion on Internet gambling protection tools, gambling habits and course, gambling problems, cognitive distortions, and gambling account information.

Then, all participants were requested to gamble at their favorite gambling website, on their favorite type of gambling, in their usual way (as if they were at home) in a quiet room specifically dedicated to the study. The room did not contain any gambling-related cues or other stimuli that might promote gambling. To be as naturalistic as possible, participants gambled with their own gambling account and their own money. The gambling session could last up to 3 h, and there was no minimum duration defined *a priori*. The screen was video recorded to be able to monitor all bets made during the gambling session. Participants were advised of this specific feature of the assessment beforehand, and have expressly consented for this. Participants were instructed at the beginning of the session that they could gamble as long as they want and just had to inform the interviewer when they wanted to end the session. If participants wanted to smoke, they had to leave the room (as the experimental room was in a hospital).

In the experimental conditions, a defined amount of money was given to the gambler during the gambling session with a bank e-card system to simulate a wagering inducement. Participants were not aware that they would receive this inducement. The time when the inducement was given was defined as the middle of the gambling session, whose duration was estimated equal to the mean duration of the session declared by the gambler in the pretest interview (and maximum after 1:30 of gambling). In the control condition, the participants received no wagering inducement.

Finally, a post-test interview was conducted at the end of the gambling session to collect the following information: subjective impact of the wagering inducement (for gamblers in the experimental conditions), cognitive distortions, enjoyment of gambling, loss of control, and gambling account information.

### Measures

We used a combination of quantitative and qualitative measures. Qualitative measures were used to collect the subjective perspective of the participants regarding Internet gambling protection tools in general (pretest) and the impact of the experimental wagering inducement on their gambling behaviors (post-test).

#### Sociodemographic Characteristics

We collected data on age, sex, living conditions, education level, and employment status during the pretest only.

#### Gambling Habits and Course

During the pretest only, we collected data on the age of gambling initiation, gambling habits (types of game, frequency, etc.) and motives for gambling. Moreover, participants were asked to indicate how much money and time they usually spent during a gambling session.

#### Previous Experience With Wagering Inducements

During the pretest only, participants were requested to report on their previous experience with wagering inducements (whether they previously received some, the usual amount of money) and their opinion (qualitative data) about the limitation of wagering inducements as a possible Internet gambling protection tool (interest in reducing the risks of problem gambling, view of the operator if he/she implemented this measure).

#### Gambling Problems

During the preselection questionnaire, participants completed the PGSI, which is a 9-item self-report questionnaire derived from the Canadian Problem Gambling Index originally developed by Ferris and Wynne (28). The total score indicates the status of the gambler: non-problem gambler (score 0), low-risk gambler (score 1–2), moderate-risk gambler (score 3–7), and problem gambler (score 8+). In the present study, the result of the PGSI was only used for eligibility and to define two categories of interest: at-risk gamblers (ARGs) were those with a moderate risk (score 3–7), and recreational gamblers (RGs), including both non-problem gamblers and low-risk gamblers, had a score of 0–2. Moreover, during the post-test, they completed a 10-point Numerical Rating Scale (NRS) to assess their feeling of losing control over gambling during the experimental gambling session.

#### Cognitive Distortions

During both the pretest and post-test, participants completed the French version of the Gambling Related Cognitions Scale (GRCS) (29, 30). The GRCS is a 23-item self-report questionnaire exploring five dimensions of cognitive distortions associated with gambling: interpretative bias (GRCS-IB), illusion of control (GRCS-IC), predictive control (GRCS-PC), gambling-related expectancies (GRCS-GE), and perceived inability to stop gambling (GRCS-IS). Moreover, participants were asked to estimate their probability of winning the next gambling session (percentage) at the pretest and post-test.

#### Enjoyment of Gambling

During the post-test, participants were requested to estimate their level of enjoyment of gambling during the experimental gambling session using a 10-point NRS.

#### Gambling Account Information

Information gathered from the participant's gambling account history was used to ensure eligibility. Moreover, as the screen was video recorded, we were able to monitor precisely all the gambling actions performed during the experimental gambling session, so that an objective and prospective record of measures of gambling (money wagered and time spent gambling) was possible. Money wagered was defined as the sum of bets during the gambling session. Time spent gambling was defined as the time during which the participant wanted to continue the gambling session, whether to place bets, prepare bets, look at sports/horse race events, etc. Thus, the beginning of the session was the moment when the participant connected to his/her account, and the end was the moment when he/she indicated that he/she wanted to end the experimental gambling session.

#### Subjective Impact (Qualitative Data Collection)

During the post-test, participants in the experimental conditions were asked about the subjective impact of the wagering inducement on their gambling behavior during the gambling session.

### Data Reduction

We used the raw value of money wagered and time spent gambling during the experimental gambling session as the two main outcomes of the study to objectively estimate the impact of wagering inducements on gambling behavior.

Moreover, we used secondary outcomes to investigate the effects of wagering inducements on gambling-related cognitions, emotions, and behaviors. We computed change scores to express variations between the pretest and post-test on GRCS scores and the subjectively rated probability of winning. Two variables (enjoyment of gambling NRS and loss of control NRS) were used as is. Finally, we created two binary variables to estimate whether the pattern of betting (money wagered and time spent gambling) during the experimental gambling session conformed to the participant's usual gambling behavior outside the laboratory. These variables were determined by comparing the objective money wagered and time spent gambling during the gambling session (gathered from the gambling account information) collected in the post-test with the baseline money wagered and time spent gambling defined in the pretest (i.e., subjective indication from the participant of how much money and time they usually spend during a gambling session).

For qualitative data, reported quotes are French excerpts of the participants' responses, translated into English.

### Statistical Analysis

We first described all variables by their number and percentage for categorical variables and by their mean and standard deviation for quantitative variables. We also graphically described the dispersion of the two main outcomes with box plots, including the median, the first and third quartiles, the non-outlier range, and the identification of outliers and extremes, to observe the relative heterogeneity of the distribution in each condition. The normality of quantitative variables was tested using the Shapiro-Wilk test, and transformations were applied whenever needed.

We then conducted a series of independent three-way ANOVAs, Poisson's regressions or logistic regressions, depending on the distribution and type of the outcome variable. These analyses were performed to compare the four experimental conditions with the control condition on the main and secondary outcomes (or their transformed equivalents), taking into account the stratification variables, i.e., the status of the gambler (recreational or at-risk), and the type of preferred gambling activity (pure chance games, skill and chance bank games and skill and chance social games). The analyses included both the effect of the condition, the effects of the stratification variables and the interaction between them. When the interactions were not significant, they were removed from the final models. When an effect was significant, pairwise comparisons were performed with Dunnett's tests only for the significant effects, by comparing each experimental condition to the control condition and controlling for the Type 1 experimentwise error.

All statistical analyses were performed using SAS® software version 9.4 (SAS Institute Inc., Cary, NC, USA).

### Ethics

The participants were informed about the research and gave their written informed consent prior to their inclusion in the study. As the procedure involved that participants, including at-risk gamblers, gambled with their own funds, several safeguards were discussed with the ethics committee and put in place to ensure financial safety of the procedure for participants. These safeguards were:

- A maximum bet limit defined for each participant before the gambling session (equal to 4 times the amount that the participant declared to bet on average per gambling session in the pretest, minus the amount of the inducement if applicable); the protocol provided the session to be stopped if the participant reached this limit.
- A maximum gambling duration of 3 h; the protocol provided the session to be stopped if the participant reached this limit.
- A compensation fund in order to partially reimburse participants for losses incurred during the experimental gambling session, when they reached a certain amount. We indemnified participants if the losses reached at least the amount that the participant declared to bet on average per session of gambling during the pretest, up to the amount lost beyond this limit (minus the amount of the inducement if applicable).
- The *a posteriori* exclusion of participants who have bet more than € 2,000 during the experimental gambling session. If this threshold was reached, the protocol provided for the session to be stopped and the participant's data not to be used in the data analysis.

As 2 of these safeguards relied on the amount of the average bets per gambling session declared by the participants, and to ensure this amount was not biased, we checked the relevance of this subjective estimate based on objective data gathered from the participants' accounts: in the event of a difference of more than 20% between the declared amount and the objective amount, the amount was re-evaluated in agreement with the participants.

Participants were not aware of these safeguards during the procedure in order not to bias their gambling behavior during the gambling session, and were only informed at the end of their participation if applicable. Moreover, although they were aware of the overall process, they were not informed beforehand that they would receive (for experimental conditions) a wagering inducement. This choice was made in order not to bias the gambling behavior during the experimental session. At the end of the post-test interview, participants were debriefed in order to investigate, after having received knowledge about the entire procedure and being aware of the safeguards (if applicable), if they wished to maintain or withdraw their consent to participate. No participant wanted to withdraw his/her consent.

This study was approved by the French Research Ethics Committee (CPP) on January 8, 2013.

## Results

As described in Table 1, we included 171 gamblers out of the 180 expected gamblers, but the sample sizes of each group were well-balanced. No participant stopped gambling before they received their assigned inducement.

**Table 1:** Sample size of each condition according to the stratification variables (status of gambler and type of preferred gambling activity).

	**Control**	**€10**	**€50**	**€100**	**€200**	**Whole sample**
**Sample size**	55	28	29	30	29	171
**Status of gambler**						
Recreational gamblers	30 (54.5%)	15 (53.6%)	15 (51.7%)	15 (50.0%)	15 (51.7%)	90 (52.6%)
At-risk gamblers	25 (45.5%)	13 (46.4%)	14 (48.3%)	15 (50.0%)	14 (48.3%)	81 (47.4%)
**Type of preferred gambling activity**						
Pure chance games	16 (29.1%)	8 (28.6%)	9 (31.0%)	10 (33.3%)	8 (27.6%)	51 (29.8%)
Skill and chance bank games	20 (36.4%)	10 (35.7%)	10 (34.5%)	10 (33.3%)	10 (34.5%)	60 (35.1%)
Skill and chance social games	19 (34.5%)	10 (35.7%)	10 (34.5%)	10 (33.3%)	11 (37.9%)	60 (35.1%)

### Description of the Sample

#### Sociodemographic Characteristics

Participants were mainly men (78.9%), with a mean age of 38 (*SD* = 11.1). Age (*F* = 0.9, *p* = 0.47) and sex (χ^2^ = 2.8, *p* = 0.58) did not differ between conditions. The participants were mainly professionally active (69.6%), with only a small proportion being either professionally inactive (18.7%), students (7.6%), or retired (4.1%). The majority of participants had an educational level that was higher than or equal to that of a high school graduate (which corresponds to 12 years of education in France) (80.7%). Finally, approximately two-thirds of the sample lived as a couple (61.4%), and the remaining third lived either alone (31.0%) or with parents or another legal representative (7.6%).

#### Gambling Habits and Course

Participants began gambling at an average age of 15 years old (*SD* = 5.6). All the participants also had an offline gambling practice, and the large majority (94.7%) of participants were initiated into gambling through offline gambling. Among the 171 participants, 66 (38.6%) reported being introduced to online gambling through promotional offers (advertising, sign-up offers, etc.).

The gambling habits of the sample are described in Table 2. Participants mainly had mixed gambling activities, combining offline and online gambling (69.6%). Just under half of the participants played multiple games on the Internet (45.0%). They mainly participated in poker, sports betting, lotteries, horse-race betting, and scratch cards. The majority of participants (83.6%) played very regularly, i.e., once per week or almost every day. Finally, the large majority of participants reported gambling for *money* and *fun and excitement*.

**Table 2:** Gambling habits of the sample (*n* = 171).

	***N* (%)**
**Gambling activity exclusively online**	52 (30.4%)
**Gambling activity centered on only one type of game**	94 (55.0%)
**Type of gambling played online**	
Poker	72 (42.1%)
Sports betting	64 (37.4%)
Lotteries	58 (33.9%)
Horse-race betting	47 (27.5%)
Scratch cards	34 (19.9%)
Black Jack	4 (2.3%)
Slots	2 (1.2%)
Roulette	2 (1.2%)
Video poker	1 (0.6%)
**Frequency of online gambling**	
Once per month or more	28 (16.4%)
Once per week or more	96 (56.1%)
Almost everyday	47 (27.5%)
**Motives for gambling online**	
Money	104 (60.8%)
Fun and excitement	90 (52.6%)
Convenience of online gambling (compared to offline gambling)	30 (17.5%)
Strategy or competition	22 (12.9%)
Avoid loneliness or boredom	16 (9.4%)
Speed and diversity of online gambling (compared to offline gambling)	9 (5.3%)
Convivial aspect of gambling	8 (4.7%)
Escapism from worries or everyday problems	3 (1.8%)
Anonymity of online gambling	3 (1.8%)
Create another life online	1 (0.6%)

### Previous Experience With Wagering Inducements

Approximately two-thirds (67.3%) of participants declared having already received wagering inducements. When asked about the usual amount of money, the large majority declared having received wagering inducements of less than €10 (70.2%). Other participants declared amounts between €11 and €50 (21.1%), between €51 and €100 (6.1%), and between €101 and €200 (2.6%). Within the sample, opinions were mixed about the value of limiting wagering inducements as a possible Internet gambling protection tool. Indeed, a third of participants (33.9%) thought that there was no interest in the limitation of wagering inducements for reducing the risks of problem gambling, a third declared a low (11.1%) or medium (23.4%) interest, and a third declared a high (25.7%) or very high (5.9%) interest. More than half of the sample thought that wagering inducements represent an incentive to gamble (54.4%): to wager more money, to experiment with new gambling activities, to register on new gambling websites, and to return to gambling after cessation, among others. Moreover, just over half of the participants reported that they would have a good (38.24%), a very good (13.53%), or an excellent (1.76%) opinion of the operator if he/she would implement the limitation of wagering inducements.

### Description and Dispersion of the Two Main Outcome Variables (Money Wagered and Time Spent Gambling During the Gambling Session)

Table 3 describes the two main outcomes (money wagered and time spent gambling during the gambling session) according to conditions (control or experimental conditions), without taking into account the type of gambling or the status of the gambler.

**Table 3:** Description of the two main outcomes (averaged money wagered and time spent gambling during the gambling session), according to the experimental condition.

	**Control *N* = 55**	**€10 *N* = 28**	**€50 *N* = 28**	**€100 *N* = 30**	**€200 *N* = 29**
Money wagered (€)—*Mean (SD)*	17.18 (22.71)	33.15 (69.45)	37.13 (51.18)	37.27 (40.69)	40.63 (60.87)
Time spent gambling (min)—*Mean (SD)*	40.96 (46.30)	54.82 (55.82)	53.29 (57.73)	48.67 (51.77)	52.90 (50.14)

**Figure 1:**
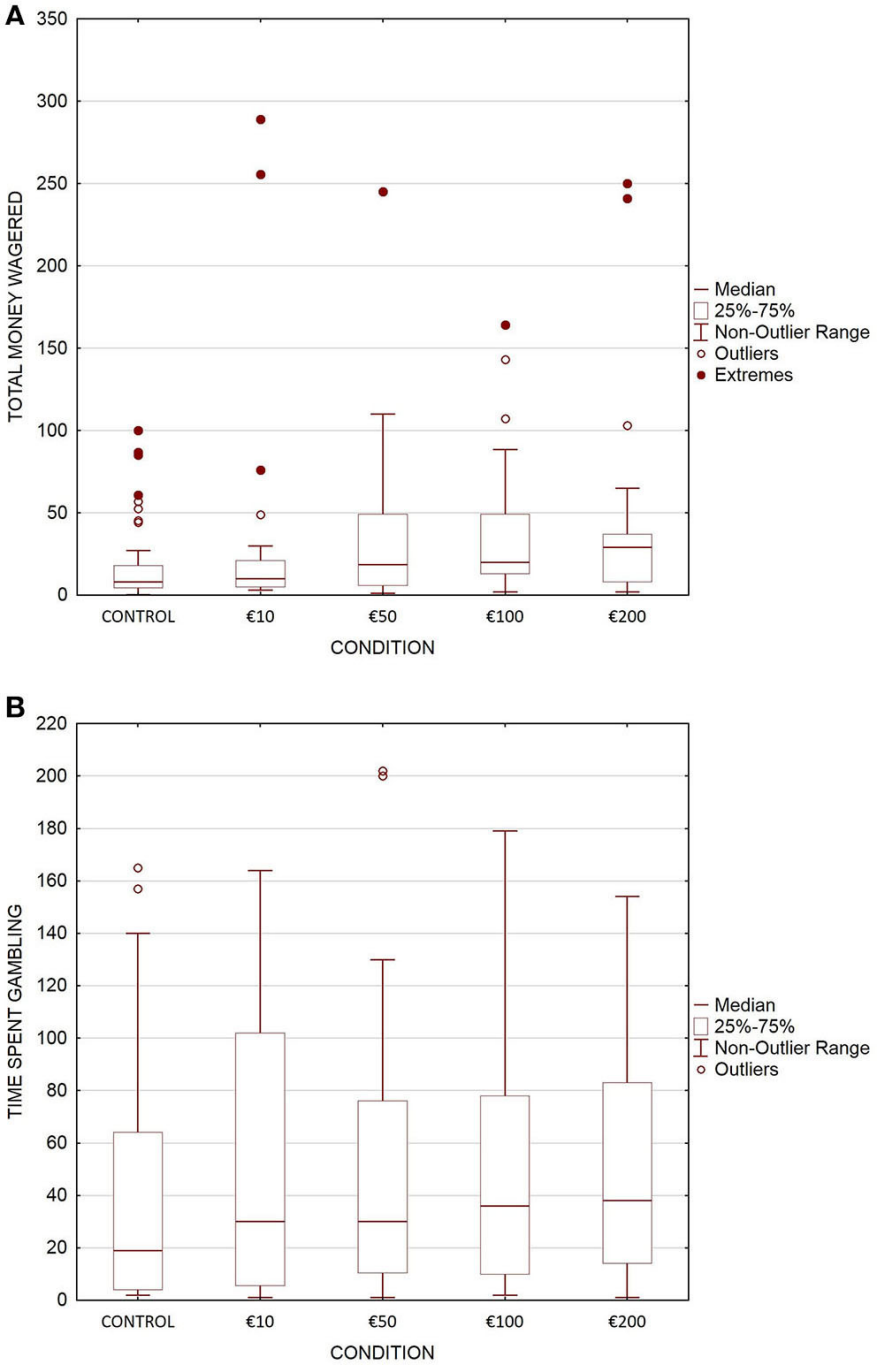
Box plots of total money wagered **(A)** and time spent gambling **(B)** during the experimental gambling session, according to the condition.

The amount of money wagered is, on average, twice as high for all experimental conditions (regardless of the amount of inducement) than for the control condition. Moreover, as depicted in Figure 1A, the dispersion of money wagered is highly variable depending on the condition. Indeed, the dispersion of the values for the control group is much smaller than that of the experimental groups, regardless of the amount of inducement. Extreme values of money wagered were predominantly observed for at-risk gamblers (of the 11 extremes identified, 9 were at-risk gamblers). Within the control group, extremes ranged from €60 to €100, whereas they ranged from €75 to €290 in the experimental conditions (and from €160 to €290 when considering only €50, €100 and €200 groups).

As depicted in Figure 1B, time spent gambling increased slightly (in the range of 8–15 min more) for the experimental conditions compared to the control condition. In contrast to the money wagered, relatively similar dispersions of values as a function of the condition are observed for the time spent gambling.

### Effect of Wagering Inducements Adjusted for the Status of Gamblers and the Type of Preferred Gambling Activity

The results of the analyses adjusted for the status of gamblers and the type of preferred gambling activity are displayed in Table 4. All interactions were not significant, so they were all removed from the final models.

**Table 4:** Results of the ANOVAs and regressions adjusted for the status of gamblers and the type of preferred gambling activity, comparing the 4 experimental conditions with the control condition on main and secondary outcome variables.

	**ANOVA effects**						**Pairwise comparisons for significant effects of the inducement conditions with Dunnett's tests^‡^**
	**Inducement**		**Status of gambler**		**Type of gambling**		
	***F***	***p*-value**	***F***	***p*-value**	***F***	***p*-value**	
**Money wagered**	3.47	**0.0095**	14.26	**0.0002**	0.18	0.8320	a^NS^; b^NS^; c^***^; d^***^
**Time spent gambling**	1.04	0.3893	6.20	**0.0138**	107.60	**<0.0001**	NA
**Change score GRCS_GE**	4.01	**0.0039**	0.05	0.8189	4.62	**0.0111**	a^***^; b^***^; c^NS^; d^NS^
**Change score GRCS_IS**	0.51	0.7255	0.02	0.8917	0.09	0.9143	NA
**Change score GRCS_IC**	0.81	0.5191	1.01	0.3169	3.34	**0.0378**	NA
**Change score GRCS_PC**	1.43	0.2267	0.91	0.3420	0.43	0.6502	NA
**Change score GRCS_IB**	0.90	0.4636	0.40	0.5279	0.90	0.4085	NA
**Change score subjectively rated probability of winning**	0.47	0.7596	0.08	0.7720	1.26	0.2855	NA
**Pleasure to gamble NRS**	2.27	0.0640	0.00	0.9668	0.95	0.3893	NA
			**Poisson's regression effects**				
			**Estimate**	**95% Wald confidence limits**		***p*****-value**	
**Loss of control NRS**							
**Inducement**						**<0.00001**	
€10 vs. control			−0.1818	−0.6713	0.3076	0.4665	
€50 vs. control			0.1792	−0.2555	0.6138	0.4191	
€100 vs. control			0.2459	−0.1713	0.6630	0.2480	
€200 vs. control			0.8263	0.4668	1.1858	**<0.0001**	
**Status of gambler**							
ARGs vs. RGs			0.9266	0.6331	1.2201	**<0.0001**	
**Type of gambling**						**<0.0001**	
Skill and chance bank games vs. pure chance games			−0.0444	−0.4463	0.3575	0.8285	
Skill and chance social games vs. pure chance games			0.7133	0.3623	1.0643	**<0.0001**	
			**Logistic regression effects**				
			**OR**	**95% Wald confidence limits**		***p*****-value**	
**Respect of the usual money wagered per gambling session (binary)**							
**Inducement**						0.1828	
€10 vs. control			1.499	0.424	5.308	0.5300	
€50 vs. control			2.097	0.526	8.358	0.2938	
€100 vs. control			1.259	0.386	4.109	0.7029	
€200 vs. control			0.444	0.158	1.252	0.1247	
**Status of gambler**							
ARGs vs. RGs			1.440	0.645	3.214	0.3732	
**Type of gambling**						0.1477	
Skill and chance bank games vs. pure chance games			1.250	0.504	3.098	0.6306	
Skill and chance social games vs. pure chance games			2.744	0.972	7.744	0.0566	
**Respect of the usual time spent per gambling session (binary)**							
**Inducement**						0.3594	
€10 vs. control			0.170	0.383	3.571	0.7824	
€50 vs. control			0.961	0.326	2.835	0.9432	
€100 vs. control			0.407	0.153	1.084	0.0722	
€200 vs. control			0.836	0.294	2.379	0.7376	
**Status of gambler**							
ARGs vs. RGs			1.152	0.569	2.334	0.6940	
**Type of gambling**						**0.0265**	
Skill and chance bank games vs. pure chance games			0.701	0.312	1.575	0.3900	
Skill and chance social games vs. pure chance games			2.402	0.937	6.158	0.0681	

*NRS = 10-point Numerical Rating Scale; Change scores: variations between pretest and post-test*.

*p-value in bold: significant effect (p < 0.05)*.

‡ *a. €10 vs. control; b. €50 vs. control; c. €100 vs. control; d. €200 vs. control. NS, non-significant*.

* *p < 0.05*;

** *p < 0.01*;

*** *p < 0.001*.

*NA, not applicable (no effect of the inducement condition)*.

*ARGs, at-risk gamblers; RGs, recreational gamblers*.

*Pure chance games: lottery and scratch cards; skill and chance bank games: horserace and sports betting; and skill and chance social games: poker*.

Regarding the stratification variables, a significant effect of status of gambler was demonstrated on the two main outcomes (money wagered and time spent gambling), with at-risk gamblers wagering more money and spending more time gambling compared to recreational gamblers, as expected. A significant effect of type of preferred gambling activity was also demonstrated on time spent gambling, with a gradient of time spent gambling from pure chance games (lowest length) to skill and chance social games (highest length) (each type differed significantly from the others in pairwise comparisons). Money wagered did not differ according to type of preferred gambling activity.

Regarding the two main outcomes, an effect of wagering inducements on money wagered has been evidenced. The pairwise comparisons indicated that this effect was significant from the amounts of €100, and there was a trend toward significance (*p* = 0.07) for amount of €50. On the contrary, no effect of inducement on time spent gambling was evidenced.

Regarding the secondary outcomes, a significant effect of inducement was demonstrated on the change score of the “gambling expectancies” subscale of the GRCS (GRCS-GE). Indeed, in the control condition, the change score of the GRCS-GE was negative (mean value: −1.79), which indicated that gambling expectancies decreased during the gambling session. In the experimental conditions (with inducement), the mean value ranged from −1.57 to +0.43, which indicated that gambling expectancies decreased less or even increased after the gambling session. Pairwise comparisons indicated that this effect was significant for amounts of €10 and €50, and there was a trend toward significance for amount of €200.

Moreover, a significant effect of inducement was also demonstrated on the change score of the loss of control NRS. The loss of control rating was low (under 1) for the control group and the €10 group and higher for other experimental conditions (1.18–2.34). Pairwise comparisons indicated that the effect of inducement on the loss of control NRS was significant for amounts of €200.

### Subjective Impact of Wagering Inducements on the Gambling Session

Of those who had a wagering inducement during the gambling session (experimental conditions), regardless of its amount, just over half of the sample reported that the inducement had an impact on their gambling practice (50.4%), the majority of whom found this effect to be high (32.8%) or very high (25.9%). The majority of gamblers who reported that the inducement had no impact on their gambling practice were from the €10 group (35.1%). Examples of impacts spontaneously reported by the participants were “*I took more risks after receiving the bonus*” (at-risk gambler of sports betting, randomized into the €10 group), “*I bet more than I originally planned and I used gambling options that I do not usually use*” (recreational gambler of lotteries, randomized into the €100 group), “*I played on more expensive tables than usual*” (recreational gambler of poker, randomized into the €50 group), and “*I wagered during the session what I usually wager in a month*” (at-risk gambler of horse betting, randomized into the €200 group).

## Discussion

The objective of the present study was to compare the impacts of different levels of wagering inducements on objective (money wagered, time spent gambling) and subjective (cognitions, emotions) gambling-related outcomes to those of no inducements in a control group.

Regarding money wagered, a significant effect of wagering inducements was demonstrated from the amount of €100, with twice the money wagered compared to the control condition. This is in favor of an effect of wagering inducements on money wagered. A lack of power may explain why we were not able to demonstrate any significant differences (or only trends) for lower amounts.

Moreover, observed values of money wagered were very scattered for experimental conditions, regardless of the amount of inducement, contrary to the control condition. This is an interesting result *per se*, which may indicate that individuals who have received a wagering inducement have very heterogeneous gambling behaviors, which can lead to extreme expenses. We noted that extreme values of money wagered were predominantly observed for at-risk gamblers and that higher extreme values were observed for higher amounts of wagering inducements (€50–€200). In our sample, the large majority of gamblers reported gambling for money, which is consistent with previous literature on gambling (31–33). In particular, several structural equation modeling analyses identified that financial motives were central to explaining paths to gambling problems (34, 35). This may explain why at-risk gamblers seem to display more extreme responses to incentives.

Regarding the time spent gambling, no effect of wagering inducements was demonstrated. Contrary to money wagered, there did not seem to be variability in dispersion across conditions. As stated in the introduction, wagering inducements are supposed to encourage betting, especially to induce an immediate sale (17). Thus, it is not surprising that gamblers in the experimental conditions are more prone to gambling more money than more time.

Along with the effect of wagering inducements on money wagared, several effects of subjective outcomes were evidenced. More specifically, experimental inducements, from amounts as low as €10, seemed to prevent gambling expectancies from decreasing during the experimental gambling session, as was observed in the control group. According to expectancy theory, the decision to perform certain behaviors is related to the anticipation of an expected outcome of these behaviors (36). This theory was initially developed to explain the relation between motivation and work but has been largely adapted for addictive behaviors, especially substance-related, alcohol-related and gambling disorders, given the reinforcing effects expected from these addictive behaviors (37–40). The concept of gambling expectancies largely overlaps that of gambling motives, as expectancies represent the expected effects that motivate gambling initiation and maintenance despite persistent losses (30). In a recent study, Barrada et al. found that reward (and punishment) sensitivity was related to gambling behavior only through gambling motives, especially the affect regulation factor that corresponds to both positive and negative affect upregulation (41). This factor is quite similar to the GRCS-GE dimension from the GRCS, which includes gambling-related expectancies associated with both positive and negative affects [“*Gambling makes things seem better*” or “*Having a gamble helps reduce tension and stress*” (30)]. Therefore, wagering inducements hold a reward value that may have strengthened gambling expectancies during the gambling experimental session. It is important to highlight that in Barrada's study, the affect regulation factor was the strongest predictor of gambling severity (41). Although we were unable to demonstrate a significant inducement^*^status of gambler interaction in this single gambling session study, one may hypothesize that the repetition of wagering inducements in the long term may lead to a chronic increase in gambling expectancies and secondarily to gambling problems.

Moreover, an effect of wagering inducements on the feeling of losing control was revealed for amounts of €200. The loss of control is one of the key symptoms of addiction (42). The fact that wagering inducements lead to an increased perception of losing control over gambling behavior is consistent with the subjective impact reported by gamblers who were in the experimental conditions. On the scale of a gambling session, this effect on the loss of control could lead gamblers to experiment with more risky or unusual gambling options or to bet more than intended. Such behaviors may induce more damage, particularly for excessive gamblers.

According to our sample, wagering inducements are widespread, as the large majority of participants have already received some. The mixed opinion of gamblers about the possible limitation of wagering inducements was not surprising. Indeed, according to the qualitative study from Thomas et al., gamblers usually have a positive opinion of wagering inducements, especially in online gambling (19). In this study, and more specifically for younger men, those with low socioeconomic status and at-risk or problem gamblers, participants reported that such incentives represent benefits in the short term that they perceive as harmless free money. In our study, we demonstrated that wagering inducements increased gambling expectancies and loss of control, even on the scale of a single gambling session. As stated above, such emotional impacts may have long-term effects that could secondarily induce or exacerbate gambling problems. Such long-term risks are quite minimized by gamblers, especially at-risk gamblers (19). Finally, we can highlight that, contrary to what may have been expected intuitively, the enjoyment of gambling was not significantly accentuated in experimental conditions with wagering inducements.

The findings of this study may be considered in light of several limitations. *First*, as stated above, the limited sample size may have reduced the significant effects observed. Indeed, with the objective of being as naturalistic as possible, we decided to set up a procedure involving the presence of participants for half a day. This drastically reduced our capacity to include more participants in this exploratory study, and the results should be replicated in more ecological studies. This is planned in the framework of the EDEIN study (43), in which the impacts of wagering inducements will be assessed using behavioral tracking data in conjunction with self-reports of gambling problems, thus responding to the call for research launched by Wohl (13). However, the experimental methodology that we implemented in the present study allowed us to have access to more subjective aspects, such as gambling-related expectancies, which are of high interest in clarifying the mechanisms of wagering inducement effects. *Second*, in this study, we simulated a wagering inducement through a bank e-card system. This procedure was intended to free ourselves from the content of the advertising message going with the wagering inducement. However, such simulated incentives were not conditional upon certain gambling-related actions as they are in real conditions. This will limit the generalizability of our findings and again suggest the importance of carrying out more ecological studies. *Third*, excessive gamblers (scoring 8 or more on the PGSI) and treated gamblers were excluded from this study due to ethical reasons in relation to the procedure, including a gambling session. This may have inevitably reduced the effects of wagering inducements according to the status of gamblers. *Fourth*, participants who gambled on illegal websites, such as online slots and other casino game websites (which are forbidden in France), were not included in this study. Thus, the present results may not be generalizable to those participating in unregulated online gambling activities, which were found to be associated with the highest prevalence of excessive gambling in online French gamblers (12). However, the lack of a legislative framework for such online gambling activities provides an opportunity for more aggressive marketing practices from gambling operators, including wagering inducements programs, and future research should replicate the present study with online casino gamblers. *Fifth*, certain measure used in this study did not rely on psychometrically validated instruments, such as motives to gamble. Moreover, the GRCS was not specifically validated for a use as a state measure of gambling-related cognitions. *Sixth*, we used a between-group design rather than a within-group one, in order to take into account the potential disparity of time distribution of gambling events within a gambling session, independently of this experimental procedure. Future research may therefore investigate the effects of inducements in a before/after approach, with repeated gambling sessions to ensure the reproducibility of observed effects.

Despite these limitations, we must emphasize the strengths of this study. *First*, this study was focused on an innovative theme in the gambling literature. Indeed, despite the wealth of studies on responsible gambling, wagering inducements are rarely studied with respect to their impacts on gambling behaviors, cognitions, and emotions from an addictive perspective (15), although such findings would constitute an interesting method of informing policy regulations. The present study led to new findings using an experimental procedure that went beyond qualitative or self-reported methods used in previous studies. *Second*, the procedure was designed to be as naturalistic as possible; that is, participants gambled on their favorite gambling website in a usual way (as if they were at home) during a long-lasting gambling session, with their own gambling account and their own money. *Third*, the combination of objective and subjective data gave us access to a more in-depth understanding of the impacts of wagering inducements, rather than just focusing on their impacts on gambling behaviors. This design allowed us to highlight a potential mechanism of action of wagering inducements through the increase in gambling expectancies.

## Conclusion

This study demonstrated that wagering inducements may have effects on gamblers by increasing money wagered, gambling-related expectancies and perceived loss of control. In particular, it seems that wagering inducements could lead to extreme expenses, especially for at-risk gamblers. These findings taken together indicate that wagering inducements may hold risks for certain gamblers, especially at-risk gamblers. It seems important to implement preventive measures regarding wagering inducements from a responsible gambling perspective. An example of such measures would be that at-risk and problem gamblers should not be targeted by wagering inducements (19), which implies that they must previously be identified through an algorithm based of gambling tracking data for example. This is the aim of another research program called EDEIN (43). Beyond at-risk and problem gamblers, individuals who have implemented a self-exclusion measure should not receive such inducements even after the self-exclusion period to favor a gradual resumption of controlled gambling. Another possible measure would be to explain more explicitly to gamblers the true cost of wagering inducements, especially the play-through conditions that require the gambler to make further expenditures (20), which may limit the increase in gambling-related expectancies. Future research on the impacts of wagering inducements is still needed, especially more ecological studies based on behavioral tracking data and studies assessing the differential impacts of various incentive types.

## Data Availability Statement

The datasets presented in this article are not readily available because data generated in this study included sensitive data according to the French Data Protection Authority (CNIL), that could not be transferred to other researchers to guarantee participants' anonymity. Requests to access the datasets should be directed to Gaëlle Challet-Bouju, gaelle.bouju@chu-nantes.fr.

## Ethics Statement

This study involved human participants and was reviewed and approved by the French Research Ethics Committee (CPP) OUEST IV. The participants provided their written informed consent to participate in this study.

## Author Contributions

GC-B and JC designed the study, obtained funding, and were responsible for the project management and interpretation of data. GC-B wrote the first draft of the manuscript. MG-B included participants and provided the medical supervision of the study. AS, JL, and YD collected the data. MP performed the statistical analysis. All authors gave feedback on the first draft of the manuscript and approved the submitted manuscript.

## Conflict of Interest

GC-B, MG-B, AS, JL, YD and JC declare that the University Hospital of Nantes received funding from the gambling industry (FDJ and PMU) in the form of a philanthropic sponsorship (donations that do not assign purpose of use). Scientific independence with respect to these gambling industries is guaranteed, and this funding has never had any influence on the present work. The remaining author declares that the research was conducted in the absence of any commercial or financial relationships that could be construed as a potential conflict of interest.
